# MicroRNA-30a-5p inhibits gallbladder cancer cell proliferation, migration and metastasis by targeting E2F7

**DOI:** 10.1038/s41419-018-0444-x

**Published:** 2018-03-14

**Authors:** Yuan-Yuan Ye, Jia-Wei Mei, Shan-Shan Xiang, Huai-Feng Li, Qiang Ma, Xiao-Ling Song, Zheng Wang, Yi-Chi Zhang, Yong-Chen Liu, Yun-Peng Jin, Yun-Ping Hu, Lin Jiang, Fa-Tao Liu, Yi-Jian Zhang, Ya-Juan Hao, Ying-Bin Liu

**Affiliations:** 1 Department of General Surgery, Xinhua Hospital, Affiliated to Shanghai Jiao Tong University School of Medicine, 1665 Kongjiang Road, Shanghai, 200092 China; 2 Shanghai Key Laboratory of Biliary Tract Disease Research, 1665 Kongjiang Road, Shanghai, 200092 China; 3 Shanghai Research Center of Biliary Tract Disease, 1665 Kongjiang Road, Shanghai, 200092 China

## Abstract

Gallbladder carcinoma (GBC), the most common malignant tumour of the bile duct, is highly aggressive and has a poor prognosis. MicroRNA-30a-5p (miR-30a-5p) is an important tumour suppressor that participates in many aspects of carcinogenesis and cancer development. However, the role of miR-30a-5p in GBC development remains to be determined, as do the mechanisms underlying its effects in GBC. Using samples collected from 42 subjects with gallbladder carcinoma (GBC), we showed decreased miR-30a-5p expression in the primary lesions vs. non-tumour adjacent tissues (NATs). Decreased miR-30a-5p was associated with shorter disease-free survival (DFS) and overall survival (OS). Inhibiting miR-30a-5p expression in 2 representative GBC cell lines (GBC-SD and NOZ) increased cell proliferation, migration, invasiveness, as well as β-catenin nuclear translocation, vice versa. In nude mice, NOZ cells transfected with miR-30a-5p mimics grew slower (vs. miR-NC) upon subcutaneous inoculation, and had lower rate of hepatic metastasis upon spleen inoculation. Dual luciferase assay confirmed that E2F transcription factor 7 (E2F7) was a direct target of miR-30a-5p and antagonized the effects induced by miR-30a-5p downregulation in GBC cells. MiR-30a-5p attenuates the EMT and metastasis in GBC cells by targeting E2F7, suggesting miR-30a-5p is a tumour suppressor that may serve as a novel potential prognostic biomarker or molecular therapeutic target for GBC.

## Introduction

GBC is the most common and aggressive malignancy of the bile duct, and the worldwide incidence of the disease is increasing annually^1^. The prognosis of GBC is very poor, as the 5-year survival rate for patients with the disease is <5%^2^. Despite advances in the modalities used for GBC diagnosis and treatment, the clinical outcomes of GBC have not significantly improved because the disease metastasizes early and its diagnosis is often delayed^3^. Surgical resection is currently the only effective treatment for GBC, as therapeutic regimens capable of attenuating or preventing GBC metastasis are lacking^4^. Therefore, studies aiming to elucidate the molecular mechanisms mediating GBC initiation and progression, including the genetic and epigenetic alterations, are urgently needed. Identifying novel genes associated with GBC development and progression may enable clinicians and researchers to identify GBC-specific diagnostic biomarkers and to develop therapies capable of preventing cancer metastasis.

MicroRNAs (miRNAs) are a class of small non-coding RNA molecules ~18–25 nucleotides in length^5^. As endogenous suppressors of gene expression, miRNAs can bind directly to the 3′ untranslated regions (3′-UTRs) of specific target messenger RNAs (mRNAs) to induce mRNA degradation or repress protein translation^6^. Thus, these small molecules can function as tumour promoters or suppressors^7^. Among these miRNAs, miR-30a-5p, which is located in the chromosomal region 6q13, has been reported to be deregulated in several human cancers^8–11^. However, the pathological relevance and clinical significance of miR-30a-5p in GBC remain unknown. Thus, in this study, we explored the tumour suppressor role of miR-30a-5p in GBC cell lines. We found that the transcription factor E2F7 is a novel, direct target of miR-30a-5p in GBC and that an inverse correlation exists between miR-30a-5p and E2F7 expression mRNA levels in GBC tissues.

## Results

### miR-30a-5p expression deregulation is correlated with poor survival

**Fig. 1:**
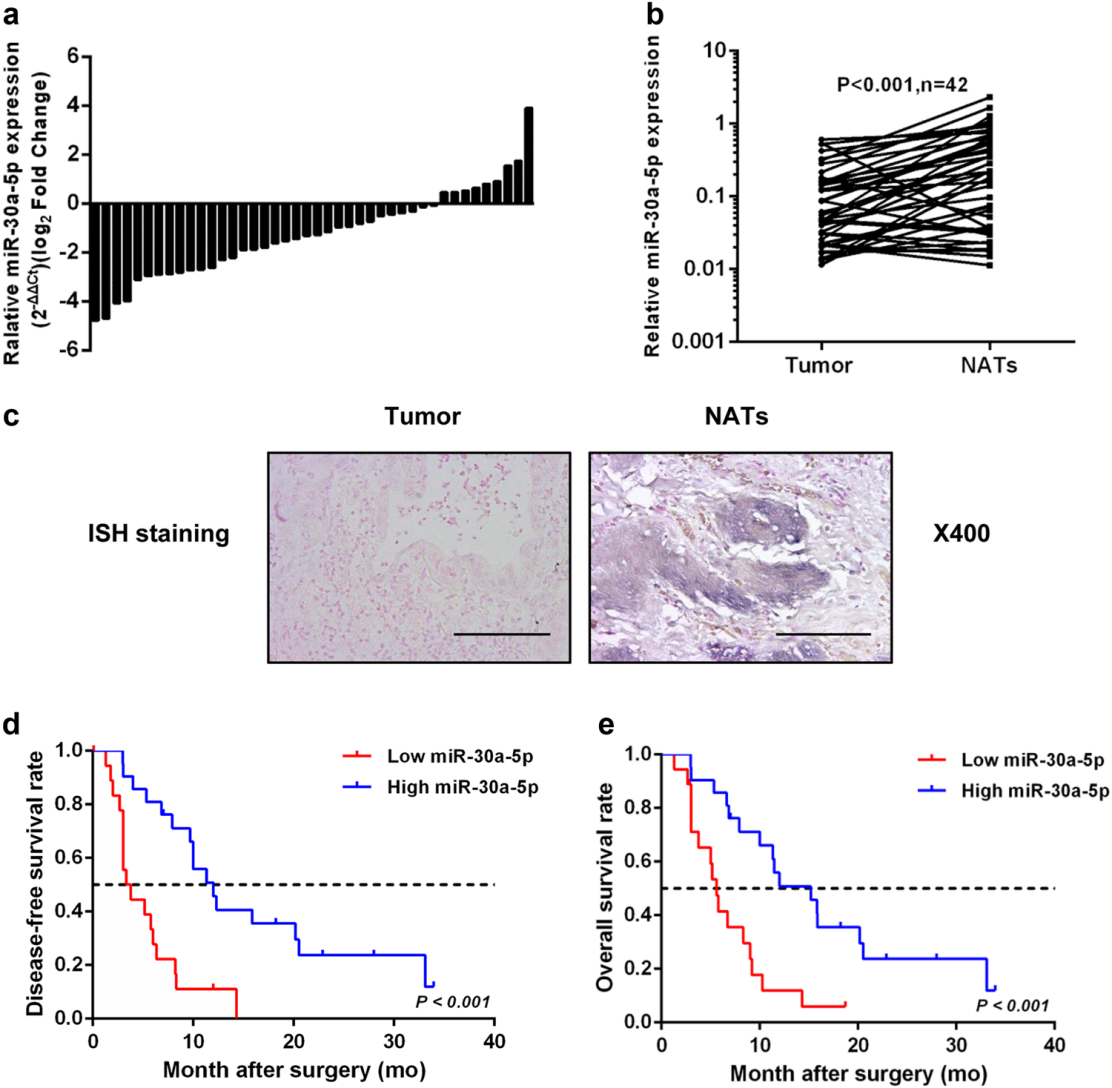
miR-30a-5p expression is significantly downregulated in GBC and is negatively correlated with patient survival. **a** Relative miR-30a-5p expression levels in 42 patients with GBC. **b** Relative miR-30a-5p expression levels in GBC tissues (Tumour) (*n* = 42) and non-tumour adjacent tissues (NATs) (*n* = 42) {*P* < 0.001). **c** Representative images of the ISH staining analyses of GBC tissues and NATs using anti-miR-30a-5p probe. **d** Kaplan–Meier curves for DFS in patients with GBC with high and low miR-30a-5p expression levels (*P* < 0.001). miR-30a-5p levels were assessed by qRT-PCR, and the median value for all 42 cases was chosen as the cutoff point with which the cases were separated into high (*n* = 21) and low (*n* = 21) miR-30a-5p expression groups. **e** Kaplan–Meier curve for OS in patients with GBC with high (*n* = 21) and low (*n* = 21) miR-30a-5p expression levels (*P* < 0.001). Scale bar = 100 μm

qRT-PCR showed lower miR-30a-5p in the primary GBC lesions vs. in the NATs in 33 out of a total of 42 cases (*P* < 0.001; Fig. 1a, b). In situ hybridisation (ISH) staining confirmed lower miR-30a-5p in the primary lesions (Fig. 1c). A Kaplan–Meier analysis showed shorter disease-free survival (DFS) and overall survival (OS) in the subjects with low miR-30a-5p (below sample median) (*P* < 0.001 vs. high miR-30a-5p; Fig. 1d, e). The subjects with low miR-30a-5p expression had larger tumour size (*P* = 0.029) and higher rate of lymph node metastasis (*P* = 0.001) (Table 1). All the clinical data suggest that miR-30a-5p may function as a tumour suppressor in the progression of GBC.

**Table 1:** Association of miR-30a-5p expression with the GBC clinicopathological characteristics

Variable	Category	Relative miR-30a-5p expression		*χ*2	*p*
		Low (*n* = 21)	High (*n* = 21)		
Age	<60	4	6	0.525	0.359
	≥60	17	15		
Gender	Male	7	7	0.000	0.628
	Female	14	14		
**Tumour size (cm)**	**<3**	**9**	**16**	**4.842**	**0.029**
	**≥3**	**12**	**5**		
Histology differentiation	Well or moderate	14	13	0.104	0.500
	Poor	7	8		
Tumour invasion(AJCC)	Tis-T2	6	4	0.525	0.359
	T3-T4	15	17		
**Lymph node metastasis**	**Yes**	**15**	**4**	**11.629**	**0.001**
	**No**	**6**	**17**		
TNM stage(AJCC)	I–II	5	4	0.141	0.500
	III–IV	16	17		

Bold values indicate stastistical significance, *P* < 0.05

### miR-30a-5p inhibits GBC cell proliferation, migration and invasion *in vitro*

**Fig. 2:**
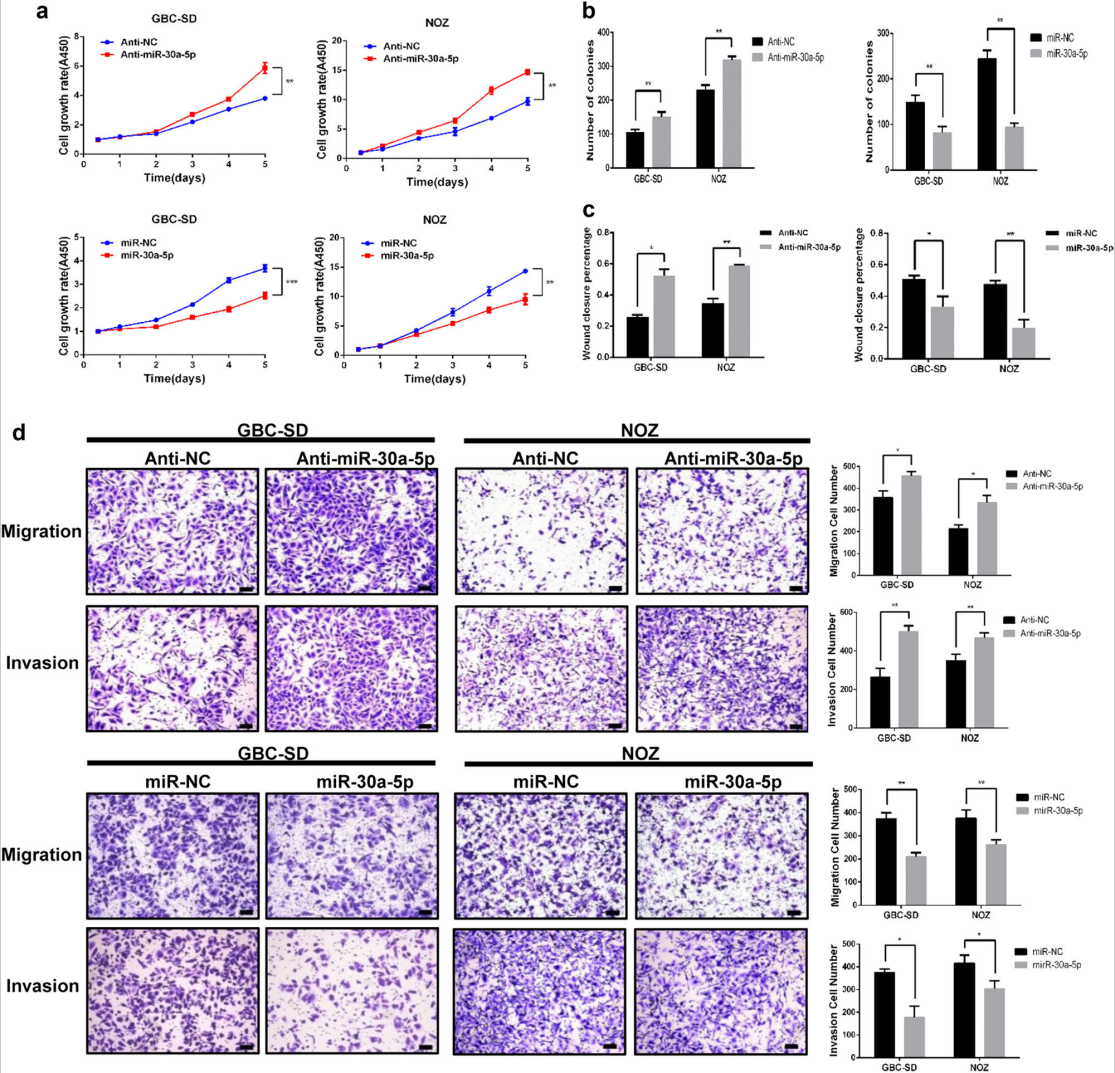
miR-30a-5p inhibits GBC cell proliferation, migration and invasion *in vitro*. **a** Cell proliferation ability was assessed in GBC-SD cells and NOZ cells infected with miR-30a-5p inhibitors or negative control miRNA inhibitors and miR-30a-5p mimics or negative control miRNA mimics. **b** Representative results of the colony formation assay in which specific cell lines were treated with anti-NC and anti-miR-30a-5p or miR-NC and miR-30a-5p. The numbers of colonies in each group were counted and are compared in the diagrams. **c** Percentage of cells that passed through the membrane and wound closure percentage. **d** Cell migration and invasion were assessed in GBC cells in which miR-30a-5p was inhibited or overexpressed. Scale bar = 100 μm. *P < 0.05, **P < 0.01, ***P < 0.001

Downregulation of miR-30a-5p in representative GBC cell lines (GBC-SD and NOZ) by transfection with miR-30a-5p inhibitor promoted cell growth and colony formation. In contrast, miR-30a-5p mimics inhibited cell growth and colony formation in both cell lines (Fig. 2a, b and Supplementary Figure S1c). The anti-miR-30a-5p increased migration and invasion ability in both GBC-SD and NOZ cells in wound healing (Fig. 2c and Supplementary Figure S1d) and transwell assay (Fig. 2d), while miR-30a-5p mimics decreased migration and invasion ability.

**Fig. 3:**
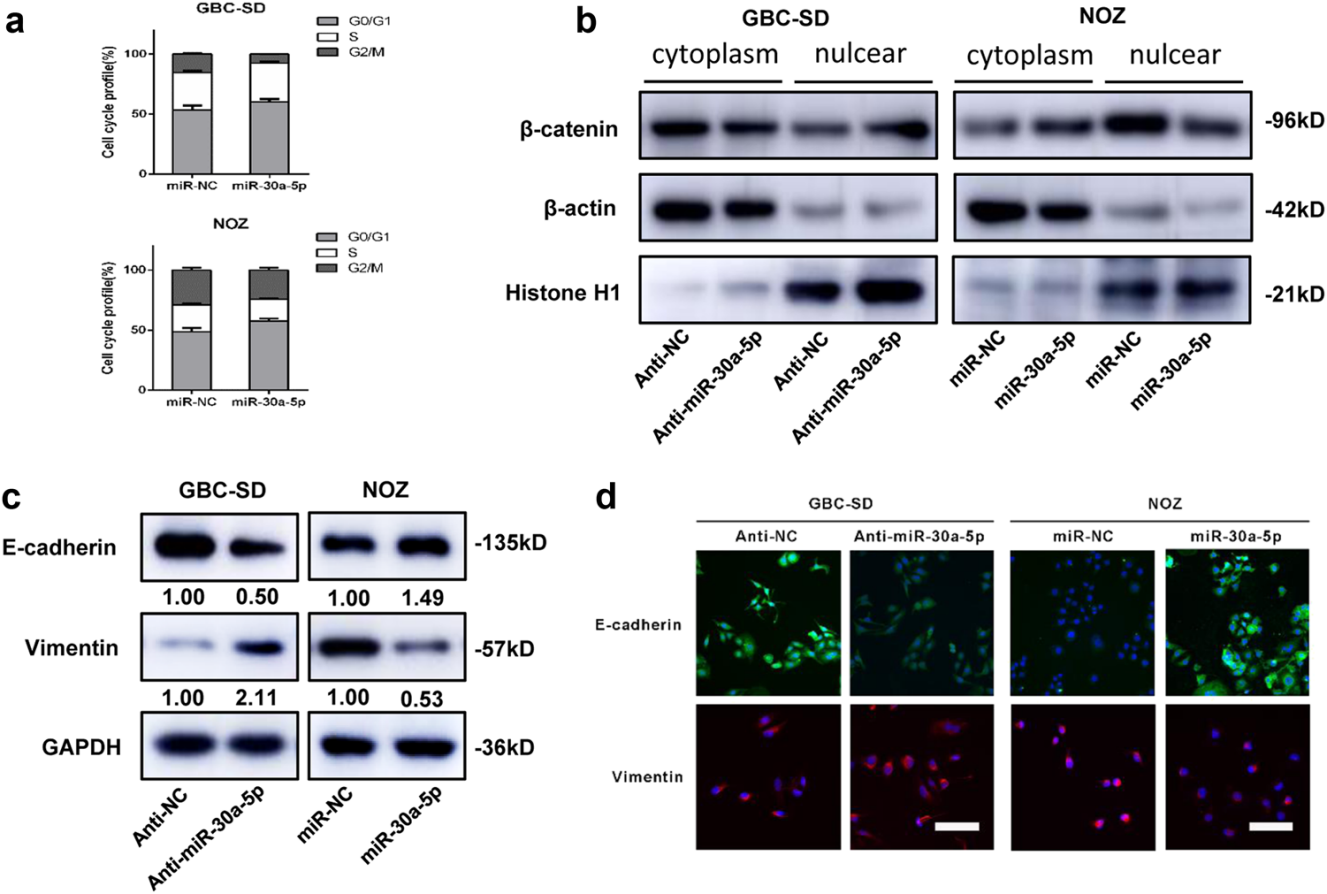
miR-30a-5p represses GBC cell cycle progression and EMT *in vitro*. **a** Representative results of the cell cycle analysis in which specific cell lines were treated with miR-NC and miR-30a-5p. **b** Subcellular β-catenin expression in indicated cells was detected by western blotting. β-actin and Histone H1 were used as a loading control. **c** E-cadherin and vimentin protein expression levels in the indicated cells were examined by western blotting. **d** E-cadherin and vimentin protein expression levels in the indicated cells were examined by Immunofluorescence. The nuclei were counterstained with DAPI. Scale bar = 100 μm

Flow cytometry showed comparable rate of apoptosis in cells transfected with the miR-30a-5p mimics vs. miR-NC (8.3 vs. 6.6% in GBC-SD cells; 2.2 vs. 1.7% in NOZ cells) (Supplementary Figure S2b). miR-30a-5p mimics blocked G1/S transition (as reflected by higher percentage of cells in the G1 phase) in both cell lines (Fig. 3a and Supplementary Figure S2a).

Western blotting and immunofluorescence assay showed that downregulation of miR-30a-5p significantly decreased the expression of E-cadherin and increased vimentin expression (Fig. 3c–d and Supplementary Figure S2c-d). Anti-miR-30a-5p increased β-catenin in the nuclear fraction and decreased β-catenin in the cytosolic fraction. In contrast, miR-30a-5p mimics decreased β-catenin in the nuclear fraction and increased β-catenin in the cytosolic fraction (Fig. 3b and Supplementary Figure S2e).

To determine the role of miR-30a-5p in GBC development, we analysed miR-30a-5p expression levels in five GBC cell lines (Supplementary Figure S1a). Remarkably, we found that the NOZ cell line, which possesses the highest metastatic potential of the five GBC cell lines analysed herein, expressed miR-30a-5p at lower levels than the other cell lines. In contrast, the GBC-SD and SGC-996 cell lines, which possess the lowest metastatic potential of the cell lines analysed herein, expressed miR-30a-5p at higher levels than the other cell lines. These results are consistent with the experiments in which a negative correlation between miR-30a-5p expression levels and lymph node metastasis was observed in the GBC tissue samples.

To determine the role of miR-30a-5p in GBC, we transfected miR-30a-5p mimics and a specific miR-30a-5p inhibitor, anti-miR-30a-5p, into GBC-SD and NOZ cells and then measured miR-30a-5p expression levels by real-time PCR (Supplementary Figure S1b).

### miR-30a-5p suppresses GBC cell proliferation, migration and invasion *in vivo*

**Fig. 4:**
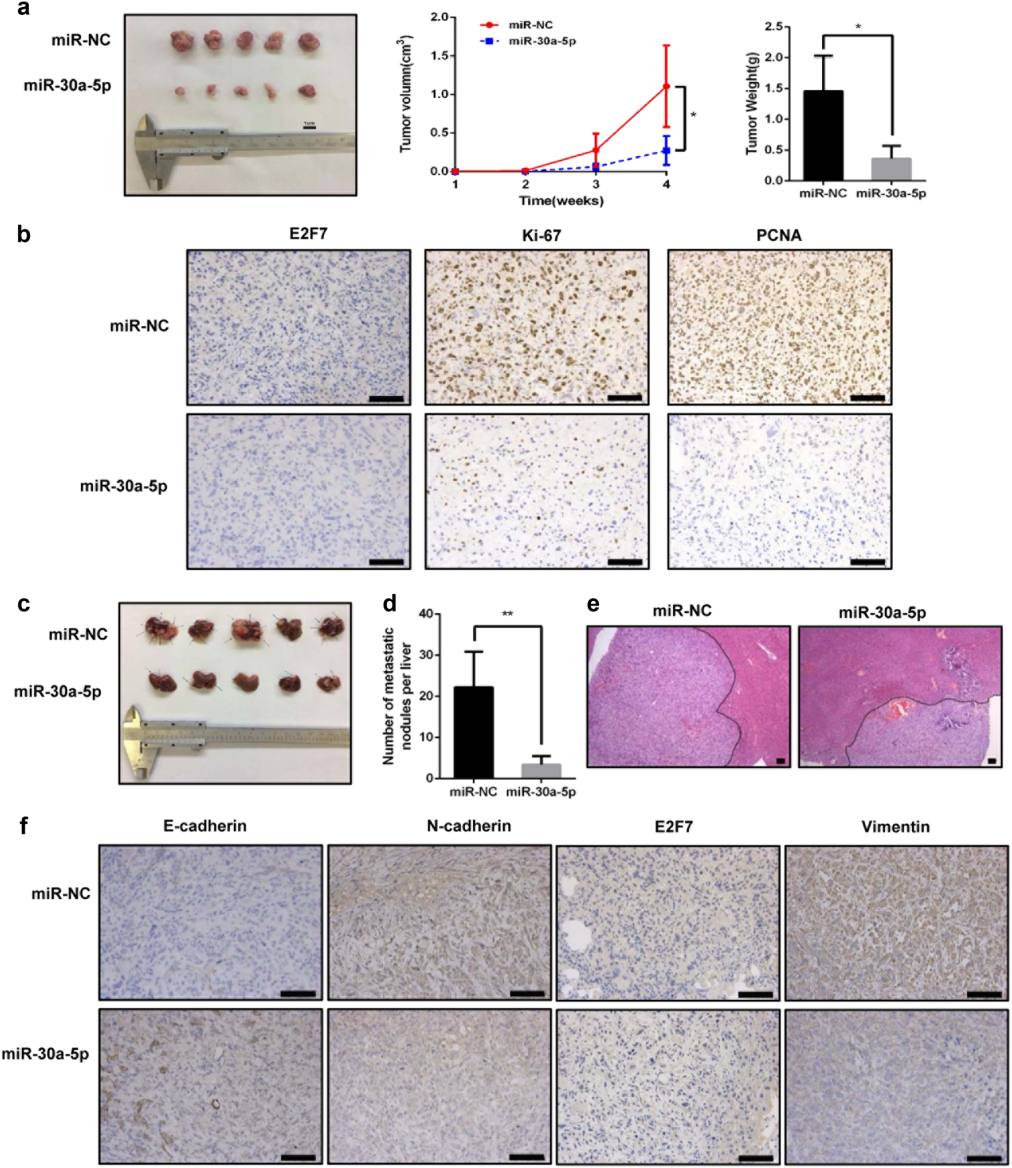
miR-30a-5p overexpression suppresses tumour growth and metastasis *in vivo*. **a** Representative example of nude mice at 3 weeks post-injection with subcutaneous xenografts of NOZ cells overexpressing miR-30a-5p (five mice per group). Quantitative analysis of xenografted tumour volumes and weights. **b** Immunohistochemical staining for E2F7, Ki-67 and PCNA in tumour specimens from local tumour tissues. **c** Representative example of liver metastases at 4 weeks after NOZ cells overexpressing miR-30a-5p were injected into the spleens of mice. **d** The number of metastatic nodules in each group was counted. **e** Representative images of liver metastasis. **f** E-cadherin, N-cadherin, E2F7 and vimentin expression in the metastatic tumour tissue samples was determined by IHC. Scale bar = 100 μm. *P < 0.05, **P < 0.01

Given that miR-30a-5p overexpression inhibited tumour growth in our *in vitro* studies, we subsequently explored the effects of miR-30a-5p on tumour growth *in vivo* by IHC, the results of which showed that tumour growth and tumour weight were significantly decreased in the miR-30a-5p-overexpression group compared with the negative control group (Fig. 4a and Supplementary Figure S1e) and that Ki-67 and PCNA expression levels in the miR-30a-5p-overexpression group were lower than those in the negative control group (Fig. 4b).

To confirm the role of miR-30a-5p in the inhibition of tumour metastasis *in vivo*, we established a liver tumour metastasis assay by injecting NOZ cells transfected with miR-30a-5p mimics into the spleens of nude mice. As shown in Fig. 4c, d, control mice displayed a higher metastasis rate than miR-30a-5p mimic-treated mice. Moreover, haematoxylin and eosin staining showed that more metastatic tumour nodules were present in the livers of the control group than in the miR-30a-5p-treated group. Consistent with these results, the IHC results showed that the expression levels of E-cadherin were increased, and the expression of vimentin and N-cadherin was decreased in the miR-30a-5p-treated group compared with the control group (Fig. 4e, f). These findings supported the hypothesis that miR-30a-5p overexpression may block EMT in cancer metastasis.

### E2F7 is a direct target of miR-30a-5p

**Fig. 5:**
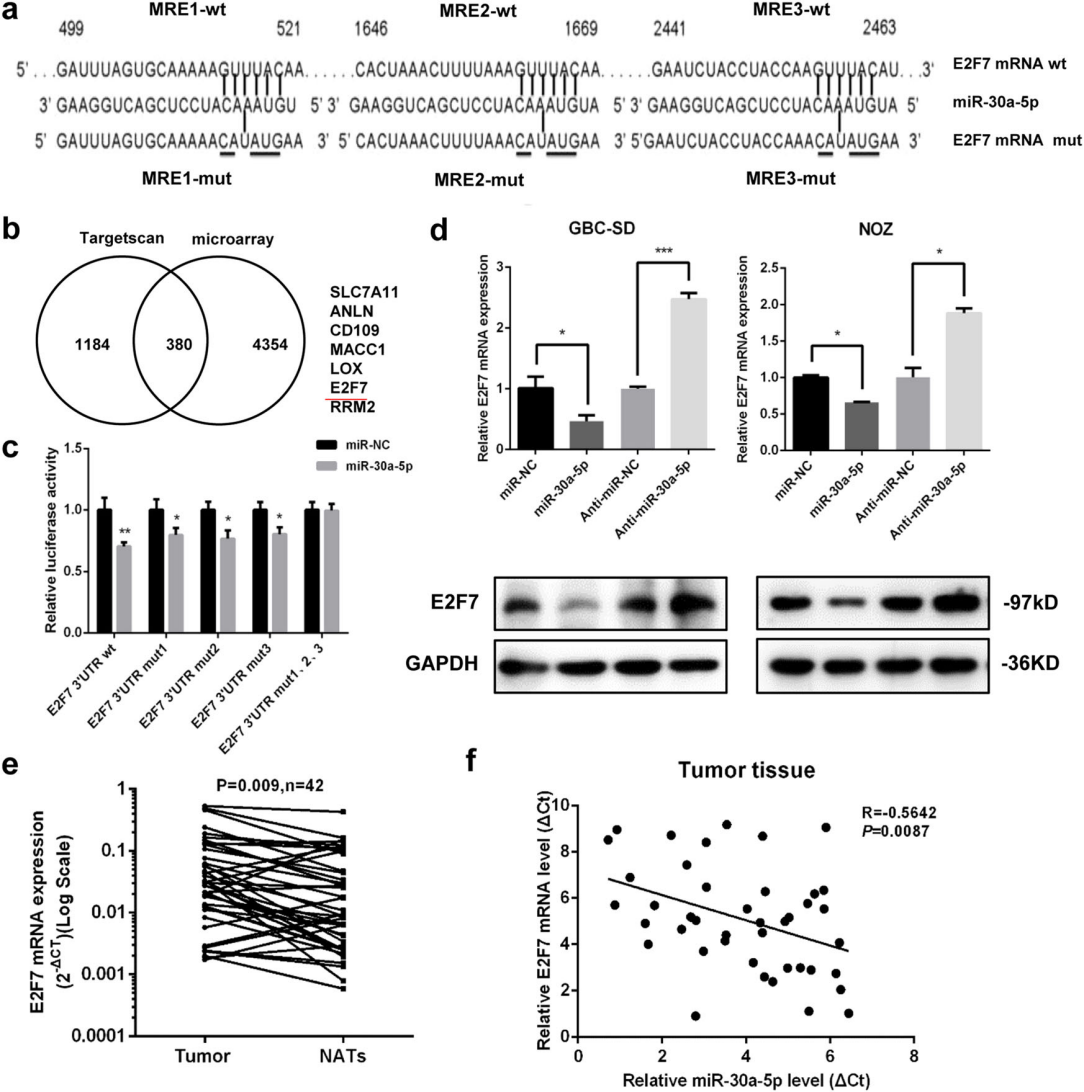
E2F7 is a direct target of miR-30a-5p in GBC cells. **a** The 3′-UTR of E2F7 mRNA contains wild-type or mutant miR-30a-5p-binding sequences. **b** Venn diagram of potential miR-30a-5p targets, as predicted by the TargetScan database and gene microarray. **c** Luciferase reporter assay was performed in 293T cells co-transfected with miR-30a-5p and pGLO-E2F7 WT or pGLO-E2F7 MΜT vectors. **d** RT-qPCR and western blot analyses of E2F7 expression levels in GBC-SD and NOZ cells transfected with miR-NC and miR-30a-5p or anti-NC and anti-miR-30a-5p. **e** E2F7 mRNA expression levels in 42 GBC tissues and matched NATs. **f** The correlation between miR-30a-5p expression levels and E2F7 expression levels was determined by linear regression analysis (*P* = 0.0087, *R* = −0.5642; Pearson’s correlation coefficient). *P < 0.05, **P < 0.01, ***P < 0.001

To elucidate the molecular mechanisms by which miR-30a-5p suppresses proliferation and metastasis in GBC cells, we searched the TargetScan database to identify the potential target genes of miR-30a-5p. We identified 380 genes between the database and the GBC tissue gene microarray. After performing a literature search (PubMed^©^, NCBI, Bethesda, MD, USA), we initially selected seven potential candidates. Given real-time PCR results showing that E2F7 has a significant downregulation after miR-30a-5p overexpression, we finally focused on E2F7 for evaluation in subsequent experiments (Fig. 5b and Supplementary Figure S3a). We then cloned E2F7 3′-UTRs containing wild-type or mutant miR-30a-5p-binding sites into luciferase reporter plasmids (Fig. 5a). Dual luciferase reporter assay showed that cells transfected with miR-30a-5p specifically inhibited E2F7-3′-UTR-WT luciferase reporter expression but not E2F7-3′-UTR-Mut reporter expression (Fig. 5c), and the western blotting and qRT-PCR results showed that miR-30a-5p repressed endogenous E2F7 mRNA and protein expression in GBC cells (Fig. 5d). These results indicate that miR-30a-5p negatively regulates E2F7 expression by directly targeting its 3′-UTR region.

### miR-30a-5p expression is negatively correlated with E2F7 expression in patients with GBC

To determine the potential clinical relevance of the miR-30a-5p-regulated E2F7 gene in GBC specimens, we determined E2F7 expression levels in 42 GBC tumour tissue specimens and NATs by qRT-PCR, the results of which showed that E2F7 mRNA expression levels were significantly upregulated in the GBC tissue specimens compared with the NATs (Fig. 5e). We subsequently performed Person correlation analysis, which showed that miR-30a-5p expression levels were significantly inversely correlated with E2F7 expression levels in GBC samples (*R* = −0.5642, *P* = 0.0087; Fig. 5f). Collectively, these results strongly indicated that the newly discovered miR-30a-5p/E2F7 axis plays a significant role in GBC progression and thus may serve as a valuable predictor of GBC recurrence and poor survival in patients with GBC.

### Gain and loss of E2F7 function abrogates and enhances the impact of miR-30a-5p on cell proliferation and metastasis, respectively

**Fig. 6:**
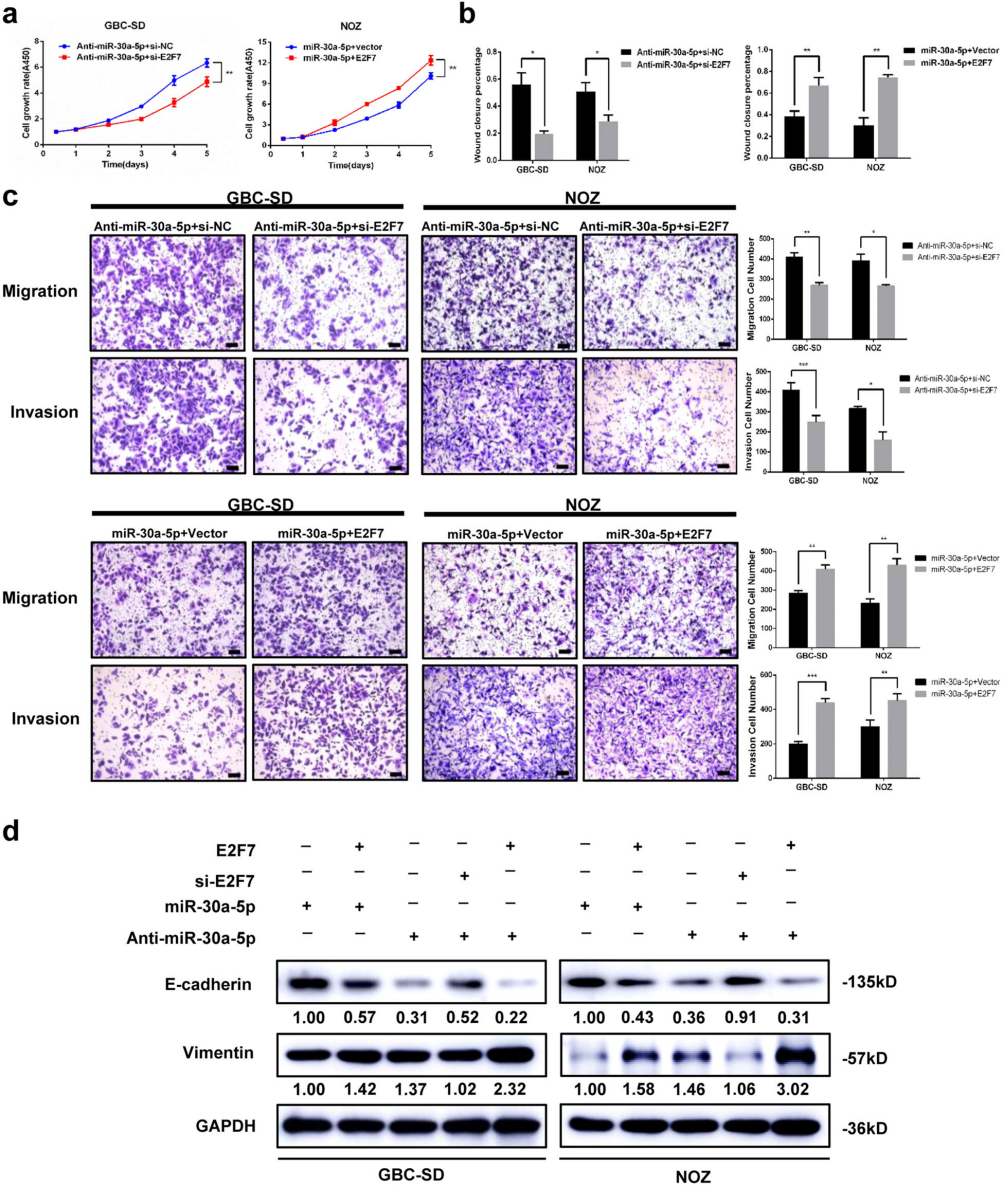
Gain- and loss-of-function studies in which E2F7 was overexpressed or depleted. Cell viability, wound closure, and cell migration and invasion ability were assayed in GBC cells transfected with miR-30a-5p and E2F7-overexpression vectors or anti-miR-30a-5p and E2F7 siRNA. **a** Cell viability; (**b**) wound closure; (**c**) migration and invasion ability. Scale bar = 100 μm. **d** E-cadherin and vimentin protein expression levels in GBC cells transfected with miR-30a-5p plus E2F7 or anti-miR-30a-5p plus si-E2F7 or anti-miR-30a-5p plus E2F7. *P < 0.05, **P < 0.01, and ***P < 0.001

To determine if E2F7 plays a pivotal role in miR-30a-5p-induced alterations in cell proliferation and metastasis, we introduced E2F7 gene or siRNA into GBC-SD and NOZ cells; and then we evaluated functional alterations in the cells. E2F7 siRNA blocked tumour cell growth, while E2F7 overexpression induced cell growth (Supplementary Figure S3b). To better understand the promoting function of E2F7 on cell proliferation, we analysed the effect of E2F7 on cell cycle. Cells transfected with si-E2F7 demonstrated more in G1 phase and blocked G1/S cell-cycle transition, suggesting that si-E2F7 inhibits GBC cell proliferation by causing G1 phase arrest (Supplementary Figure S3d). Likewise, E2F7-overexpression cells exhibited higher migration ability than vector cells, while E2F7 siRNA cells exhibited lower migration ability than negative control cells (Supplementary Figure S3c). Furthermore, western blotting assay showed that downregulation E2F7 significantly increased the expression levels of E-cadherin and decreased the expression levels of vimentin in the corresponding group compared with the control group (Supplementary Figure S3e). To further determine if E2F7 can rescue the phenotype induced by altered miR-30a-5p in the cells, we co-transfected GBC cells with either miR-30a-5p together with pCMV-E2F7, or anti-miR-30a-5p together with si-E2F7. The results showed that restoring E2F7 expression partially abrogated the reductions in proliferation and colony formation induced by miR-30a-5p in NOZ cells (Fig. 6a and Supplementary Figure S4a-b). Moreover, restoration of E2F7 expression significantly reversed the inhibitory effects of miR-30a-5p on cell migration and invasion (Fig. 6b, c and Supplementary Figure S4c). In contrast, inhibiting E2F7 expression restored the effects of miR-30a-5p on GBC cell proliferation and colony-formation capacity (Fig. 6a and Supplementary Figure S4a-b). In addition, inhibiting E2F7 expression also abrogated the stimulatory effects of anti-miR-30a-5p on GBC cell migration and invasion (Fig. 6b, c and Supplementary Figure S4c). We subsequently analysed the expression levels of several protein candidates involved in EMT by western blotting, the results of which showed that E-cadherin and vimentin protein expression levels were affected by the ectopic expression or knockdown of miR-30a-5p and that these effects were partially attenuated by the re-introduction or inhibition of E2F7, respectively (Fig. 6d). These findings support the idea that E2F7 plays an important role in the mechanisms underlying the tumour-suppressive functions of miR-30a-5p in GBC.

## Discussion

GBC carcinogenesis is multifactorial and is driven by genetic and epigenetic events^12–14^. Increasing amounts of evidence indicate that miRNAs are dysregulated in various cancers^15, 16^ and can function as tumour suppressors or promoters^17, 18^. To date, numerous studies have unveiled a number of miRNA signatures in GBC^4, 19, 20^, but the exact role of miRNA dysregulation in the pathogenesis of GBC has remained unclear. MiR-30a-5p was chosen as the subject of this study, which showed that miR-30a-5p plays an important role in GBC initiation and progression. We found that miR-30a-5p expression was downregulated in GBC tissues compared with normal gallbladder tissues. Moreover, we found that lower miR-30a-5p expression was significantly correlated with unfavourable clinicopathological characteristics, and shorter DFS and OS in patients with GBC, which strongly suggests that miR-30a-5p may serve as a novel diagnostic and prognostic biomarker for GBC.

MiR-30a is a member of miR-30 family, which also includes miR-30b, miR-30c, miR-30d, and miR-30e. As a group of tumour suppressors, the miR-30 family has been reported to be downregulated in many human cancers, including colorectal cancer^21^, lung cancer^22, 23^, thyroid cancer^24^, renal cell carcinoma^25^ and gastric cancer^26^. However, the precise role of miR-30a-5p in tumourigenesis was largely unknown prior to this study. Here we demonstrated that miR-30a-5p significantly inhibited GBC cell proliferation and migration and attenuated tumour growth and metastasis in xenografted mice.

EMT is a biological process by which epithelial cells lose their polarity and adhesive capabilities and gain the migratory and invasive properties of mesenchymal cells^27^. Previous studies have shown that EMT plays a pivotal role in regulating tumour invasion and metastasis in many human cancers^28–30^. Interestingly, miRNAs have been shown to be powerful regulators of EMT by participating in EMT-related signalling pathways and interacting with EMT-related transcription factors^31–33^. In this study, we found that miR-30a-5p altered the expression of EMT-related proteins, including E-cadherin and vimentin. Given the E-cadherin is an important component to maintain β-catenin in the membrane, downregulation of E-cadherin leads to increased nucleus translocation of β-catenin^34^. Our results showed that miR-30a-5p increased β-catenin in the cytosolic fraction and decreased β-catenin in the nuclear fraction. Although we lack the evidence that miR-30a-5p targets 3′-UTR of these genes, our present data indicate that miR-30a-5p downregulation may drive EMT in cancer cells, resulting in metastasis.

It is well known that miRNA can degrade and inhibit specific mRNAs by interacting with mRNA molecules at 3′-UTR of target genes^6^. Our results demonstrated that E2F7, which is known to play an essential role in regulating cell cycle progression^35^, is a novel target of miR-30a-5p and that miR-30a-5p exerts its tumour-suppressive effects in GBC cells at least in part by repressing E2F7. Moreover, we found that miR-30a-5p upregulation inhibited E2F7 expression in different GBC cell lines. However, to the best of our knowledge, little is known regarding the mechanisms regulating E2F7 expression and the relationship between E2F7 and miR-30a-5p in GBC have not previously been elucidated. Previous studies showed that E2F7 was an initiator that participated in cell cycle regulation, cell apoptosis and cell differentiation^36–38^. Interestingly, in our study, we identified E2F7 as a tumour-promoting protein, as it can induce tumour cell proliferation, invasion and metastasis, and also has the ability to rescue tumour invasive behaviour inhibited by miR-30a-5p in GBC. Thus, the distinct roles of E2F7 in the development of different cancers implicate that the tumour-promoting or suppressing signature of E2F7 relies on individual cancer types^39, 40^. It is worthwhile to further define potential targets of E2F7, which may differentially regulate cell growth and motility in divergent tumour cells. Nonetheless, our study has provided strong evidence pointing to the novel regulatory axis of miR-30a-5p/E2F7 in the development of GBC.

In summary, our study has demonstrated for the first time that miR-30a-5p plays pivotal roles in GBC proliferation and metastasis *in vitro* and *in vivo*, probably by directly targeting E2F7. Our findings have provided new insights into the mechanisms underlying GBC progression and indicate that miR-30a-5p may have potential as a novel prognostic biomarker for GBC and that the miRNA may also be useful as a therapeutic agent for the treatment of GBC.

## Materials and methods

### Patients and tissue specimens

This study was approved by the ethics committee of Xinhua Hospital, School of Medicine, Shanghai Jiao Tong University, China. All the patients provided written informed consent. The tumour specimens used herein were obtained from 42 patients with pathologically confirmed GBC who underwent radical tumour resection in the Department of General Surgery, Xinhua Hospital, from 2012 to 2015. Specimens from patients who underwent preoperative radiotherapy or chemotherapy were excluded from the study. The above specimens were used for quantitative real-time polymerase chain reaction (qRT-PCR) analysis. The clinicopathological characteristics of the patients from whom the specimens were obtained are presented in Supplementary Table 1.

### Cell lines and cell culture

The human GBC cell lines GBC-SD and SGC-996 were purchased from the Shanghai Institute for Biological Science, Chinese Academy of Science (Shanghai, China), and the NOZ, EH-GB1, and OCUG-1 cell lines were obtained from the Health Science Research Resources Bank (Osaka, Japan). The GBC-SD, NOZ, EH-GB1, and OCUG-1 cell lines were cultured in high-glucose DMEM (Gibco, NY, USA), and the SGC-996 cell line was cultured in Rosewell Park Memorial Institute (RPMI) 1640 medium (HyClone, Logan, UT, USA). All of the above media were supplemented with 10% foetal bovine serum (Gibco, NY, USA), and all of the above cell lines were cultured at 37 °C in a humidified atmosphere with 5% CO_2_.

### Quantitative real-time PCR

Total RNA was extracted from the above tissue samples or cultured cells with Trizol reagent (Invitrogen, Carlsbad, CA, USA), and cDNA was prepared from total RNA for mRNA and miRNA quantification using a PrimeScript^TM^ RT Master Mix Kit (Takara, Dalian, China) and a Mir-X miRNA First-Strand Synthesis Kit (Clontech Laboratories, Inc, USA), respectively. RNA expression was measured by qRT-PCR using SYBR® Green (Takara, Dalian, China) and a Mir-X miRNA qRT-PCR SYBR Kit (Clontech Laboratories, Inc, USA). GAPDH and U6 served as internal controls. Relative RNA expression levels were quantified with the 2^−△△CT^ method. The sequences of the primers used herein are listed in Supplementary Table 2.

### RNA oligonucleotides, plasmid construction, and cell transfection

The synthetic miRNA mimics and inhibitors and scrambled negative control miRNAs used herein were purchased from Biomics (Shanghai, China). Full-length E2F7 cDNA (GenBank accession number NM_203394) was cloned into a pCMV expression vector (LQbiotech, Shanghai, China). An empty vector was used as a control. GBC-SD and NOZ cells were seeded in 6-well plates and transfected using Lipofectamine 2000 (Invitrogen, Carlsbad, CA, USA). For these experiments, 50 nM miRNA mimic or inhibitor and a corresponding negative control were added to each well. For plasmid transfection, 2 μg of plasmids were added to each 6-well plate for 48 h, after which the cells were treated with G418 (200 μg/mL). The sequences of the miRNAs used for these experiments are listed in Supplementary Table 2.

### *In vitro* tumourigenesis assays

Cell proliferation was evaluated by Cell Counting Kit-8 (CCK-8; Dojindo, Japan), according to the manufacturer’s instructions, and the absorbance was measured at a wavelength of 450 nm by a microplate reader (Bio-Rad, Hercules, CA, USA). For the colony formation assays, GBC-SD and NOZ cells were seeded in 6-well plates, in which they were subsequently cultured for approximately 14 d. The cells were then fixed with 4% paraformaldehyde and stained with 0.1% crystal violet (Sigma, St. Louis, MO, USA), after which the total number of colonies (>50 cells/colony) of each cell line was counted.

### *In vitro* migration and invasion assays

For wound-healing assay, transfected GBC cells were seeded in 6-well plates in serum-free medium and grown to 90% confluence, after which scratch-wounds were created in their monolayers with a sterile 200-μL pipette tip. The wound area was measured 0 and 48 h after wound placement. Cell migration and invasion assays were performed using 8-μm transwell filters in 24-well plates (BD Biosciences, Franklin Lakes, NJ, USA). GBC-SD and NOZ (3 × 10^4^ or 2 × 10^4^) cells were plated in the upper chamber, whose membrane was coated with or without Matrigel (BD Biosciences, Franklin Lakes, NJ, USA), in 200 μL of serum-free medium. Five-hundred microliters of medium supplemented with 10% FBS was subsequently added to the lower chamber. The cells were subsequently allowed to incubate for 22 (GBC-SD) or 14 h (NOZ), after which the cells that had migrated to the lower compartment and were thus adhering to the lower membrane were fixed with methanol and stained with crystal violet. The cells were photographed, after which the cells in three randomly selected fields in each well were counted. The above experiments were performed in triplicate.

### Nude mouse subcutaneous and liver metastasis tumour models

Male BALB/c nude mice aged 4–6 weeks were purchased from the Shanghai Laboratory Animal Center of the Chinese Academy of Science (Shanghai, China) and maintained under standard conditions, in accordance with institutional animal care guidelines. The mice were used to assess GBC tumourigenicity and metastasis *in vivo*. Sex- and age-matched littermates served as control subjects. Briefly, NOZ cells were transfected with miR-30a-5p mimics or miR-NCs, and then the indicated cells were subcutaneously injected into the left armpit of each mouse (five mice/group). Tumour size was measured every week, and tumour volume was calculated as follows: tumour volume = 4*π* / 3 × (width / 2)^2^ × (length / 2), where the width and length are the shortest and longest axes of the tumour, respectively. For the endpoint experiments, the mice were sacrificed, and the tumours were harvested and weighed. To establish the liver metastasis model, we injected 2 × 10^6^ NOZ cells into the liver via the spleen. The mice were killed 1 month after injection, at which time the livers were harvested, and the number of metastatic nodules in each liver was counted.

### Cell apoptosis assays

The cells were cultured in 6-well plates for 48 h after miRNAs transfection. The apoptosis assays were evaluated by flow cytometry using the FITC Annexin V Apoptosis Detection Kit I (BD, San Diego, CA, USA) according to the manufacturer’s instruction.

### Cell cycle analysis

The indicated cells were seeded in 6-well plate for 48 h before being collected. The cells were then washed twice with cold phosphate-buffered saline (PBS), and fixed in ice-cold 70% ethanol overnight at 4 °C. Subsequently, the cells were incubated with 10 mg/mL of RNase and 1 mg/mL of propidium iodide (Sigma-Aldrich) for 30 min at 37 °C in the dark. Finally, the cells were analysed by flow cytometry (BD Biosciences).

### In situ hybridisation (ISH) and immunohistochemical (IHC) staining

The expression of miR-30a-5p in tissue specimens was observed by ISH using digoxigenin-labelled probes (Boster Biotech, Wuhan, China). MiR-30a-5p probe sequence was (5′–3′) CTTCCAGTCGAGGATGTTTACA. ISH was conducted using microRNA ISH Optimisation Kits (Boster, Wuhan, China) according to the manufacturer’s instruction. The hybridisation signals were visualised by BCIP/NBT and the nuclear was stained with fast red. Images were photographed with a fluorescence microscope.

IHC staining of patient tissue sections was performed using the anti-E2F7 (Santa Cruz), anti-Ki67 (Abcam), anti-PCNA (Abcam), anti-E-cadherin (Cell Signalling Technology), anti-N-cadherin (Cell Signalling Technology), anti-vimentin (Cell Signalling Technology). The sections were incubated overnight at 4 °C with the primary antibodies. After washing three times with PBS, the sections were incubated with the secondary antibodies at room temperature for 1 h. Subsequently, the sections were immersed in DAB for 5–10 min and counterstained with 10% Mayer’s hematoxylin. Images were photographed with a fluorescence microscope.

### Immunofluorescence analysis

The indicated cells were seeded in 24-well plates and incubated for 24 h before being stained. For these experiments, in which we stained the cells for E-cadherin and vimentin, we fixed the cells in 4% paraformaldehyde and then permeabilized them in 0.1% Triton X-100 at room temperature. After being blocked for 1 h with blocking solution, the cells were incubated with the appropriate primary antibodies overnight at 4 °C, after which they were incubated with FITC or Cy3-conjugated goat anti-rabbit IgG (Beyotime, China) at 37 °C before being counterstained with DAPI to visualise the nuclei. Fluorescence was imaged using a Leica microscope.

### Antibodies and western blotting

Goat anti-E2F7 antibodies were purchased from Santa Cruz (CA, USA), and rabbit anti-E-cadherin, anti-β-catenin, anti-vimentin, anti-GAPDH antibodies, anti-β-actin and anti-Histone h1 antibodies were obtained from Cell Signalling Technology (Danvers, MA, USA). The subcellular protein was extracted by nuclear and cytoplasmic protein extraction kit (Beyotime, Shanghai, China).Briefly, equal amounts of cellular protein were separated by sodium dodecyl sulfate polyacrylamide gel electrophoresis and then transferred to polyvinylidene difluoride membranes, which were immunoblotted with the appropriate primary antibodies and then incubated with HRP-conjugated secondary antibodies. The blots were subsequently visualised by chemiluminescence (Millipore, Billerica, MA, USA). GAPDH was used as an endogenous control.

### Luciferase reporter assay

A 2047-bp fragment of the E2F7 3′UTR containing three conserved miR-30a-5p-binding sites was inserted into a luciferase reporter plasmid (LQbiotech, Shanghai, China), and a synthetic E2F7 3′-UTR mutant fragment was inserted into an equivalent reporter plasmid. For the luciferase assays, HEK-293T cells were seeded in 24-well plates in triplicate and allowed to settle for 24 h. The cells were then co-transfected with 10 ng of firefly luciferase reporter plasmid and an equal amount (50 pmol) of miR-30a-5p mimics or scrambled negative control RNA using Lipofectamine 2000 (Invitrogen, Carlsbad, CA, USA). At 24 h post-transfection, the cells were assayed using a luciferase assay kit (Promega, Madison, WI, USA).

### Statistical analysis

All the data are presented as the mean ± SD and were analysed using SPSS 19.0 software. All experiments were performed in triplicate. miR-30a-5p expression levels were compared between tumour tissues and paired non-tumour tissues using paired Student’s *t* tests, and the difference in the mean expression level between the two groups was assessed by independent Student’s *t* tests. The associations between miR-30a-5p expression levels and clinicopathologic characteristics were analysed by Pearson’s *Χ*^2^ test, and survival analysis was performed with Kaplan–Meier plots and the log-rank test. *P < 0.05* was considered statistically significant.

## Electronic supplementary material

Supplementary Figure 1(TIF 3777 kb)

Supplementary Figure 2(TIF 1666 kb)

Supplementary Figure 3(TIF 3093 kb)

Supplementary Figure 4(TIF 4083 kb)

supplementary table 1(DOCX 14 kb)

supplementary table 2(DOCX 15 kb)
