# Supplementary figures and images for: MicroRNA-30a-5p inhibits gallbladder cancer cell proliferation, migration and metastasis by targeting E2F7
Source: Cell Death Dis. 2018 Mar 14;9(3):410. doi: 10.1038/s41419-018-0444-x (PMC5852001; doi:10.1038/s41419-018-0444-x)

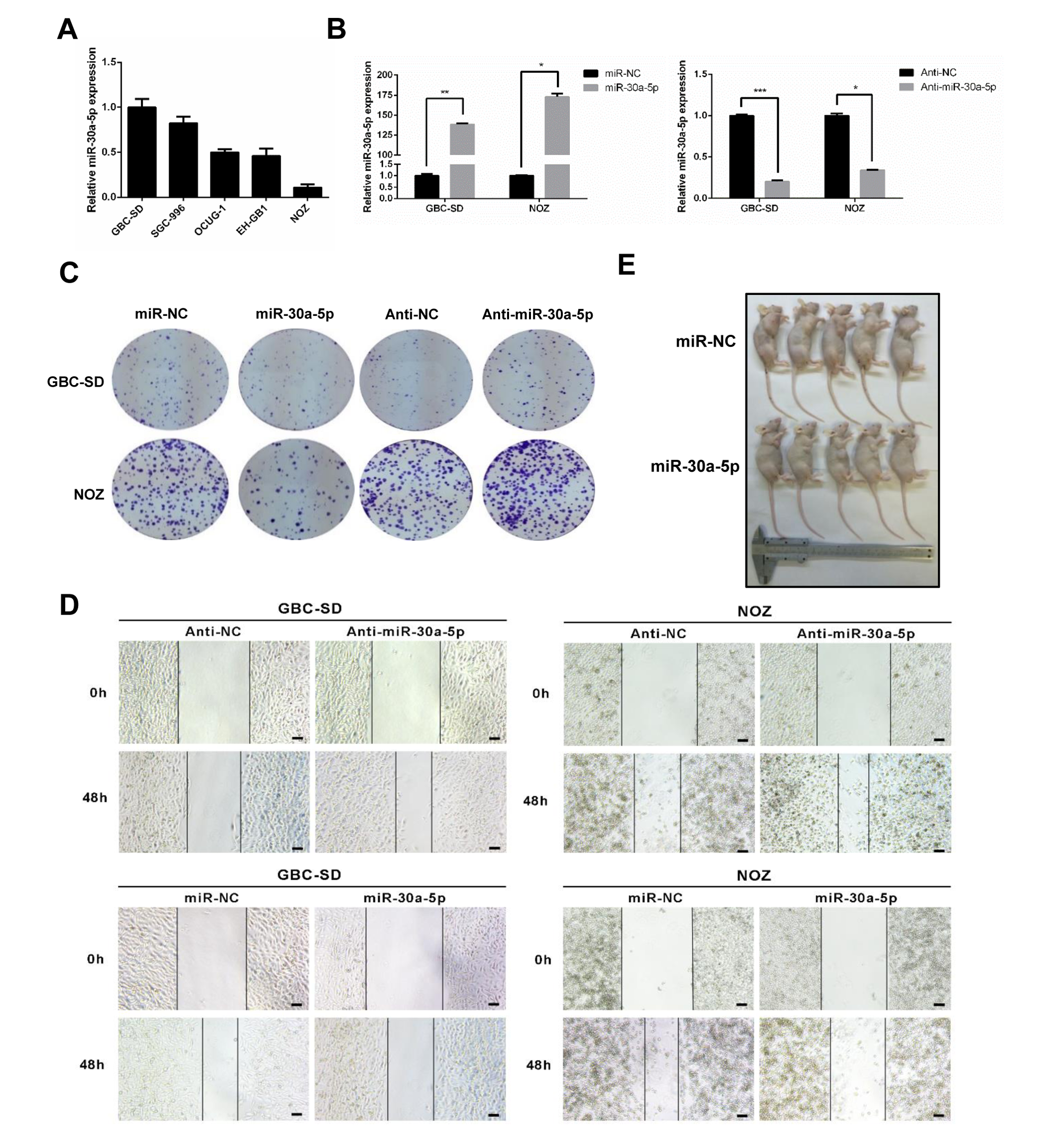

Supplement: Supplementary file 1 — Supplementary Figure 1(TIF 3777 kb) [file 41419_2018_444_MOESM1_ESM.tif]

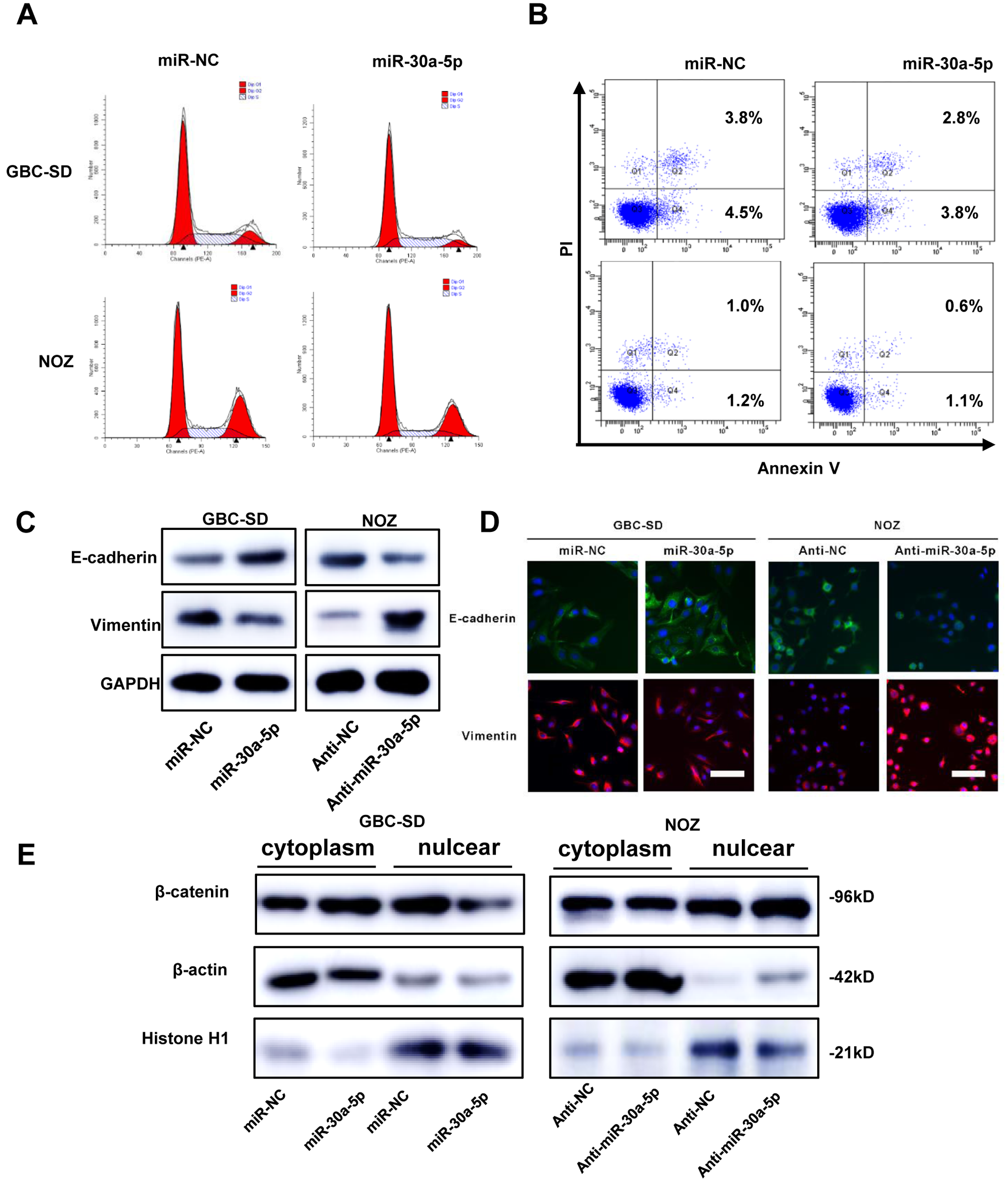

Supplement: Supplementary file 2 — Supplementary Figure 2(TIF 1666 kb) [file 41419_2018_444_MOESM2_ESM.tif]

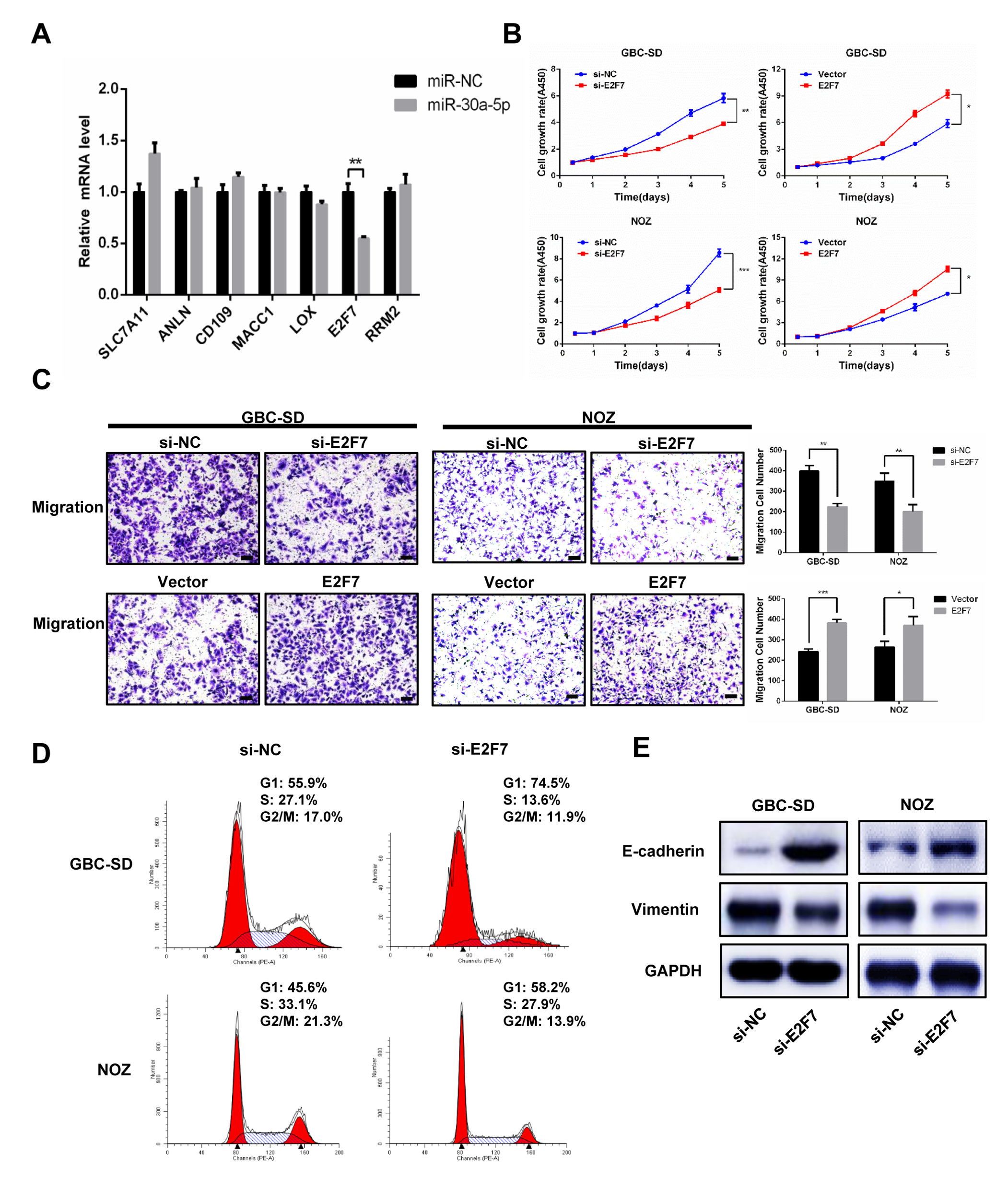

Supplement: Supplementary file 3 — Supplementary Figure 3(TIF 3093 kb) [file 41419_2018_444_MOESM3_ESM.tif]

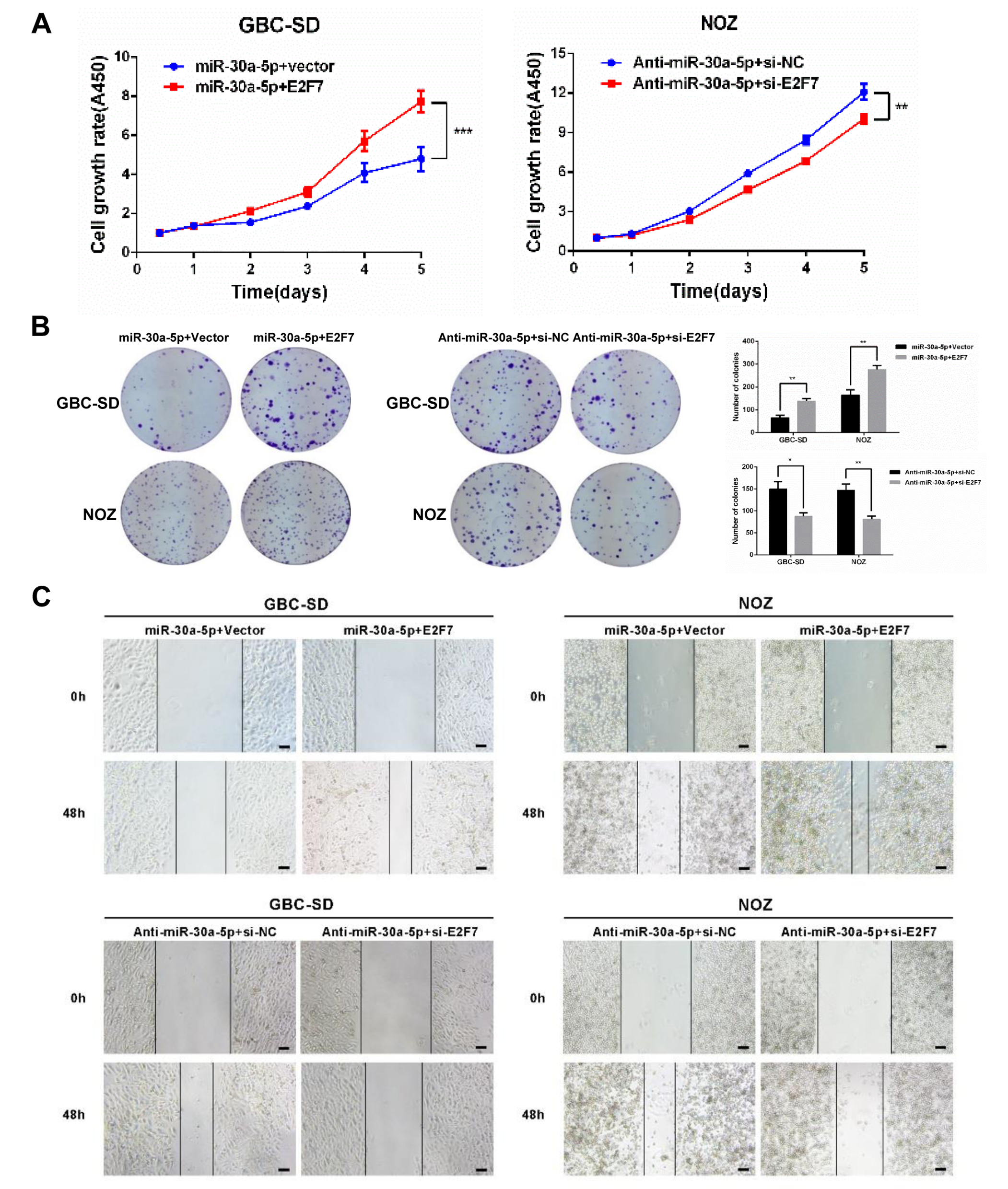

Supplement: Supplementary file 4 — Supplementary Figure 4(TIF 4083 kb) [file 41419_2018_444_MOESM4_ESM.tif]
