# Supplementary material for: MicroRNA-30a-5p inhibits gallbladder cancer cell proliferation, migration and metastasis by targeting E2F7
Source: Cell Death Dis. 2018 Mar 14;9(3):410. doi: 10.1038/s41419-018-0444-x (PMC5852001; doi:10.1038/s41419-018-0444-x)
Supplement: Supplementary file 5 — supplementary table 1(DOCX 14 kb) [file 41419_2018_444_MOESM5_ESM.docx]

| **Supplementary Table 1. Clinicopathologic characteristics of patients** | | |
| --- | --- | --- |
| Characteristic |  | Number of Patients |
| Patients |  | 42 |
| Sex |  |  |
| Male |  | 14 |
| Female |  | 28 |
| Age (years) |  | 47-84, median=67 |
| Tumour size (cm) | | 1.0-10.0, median=2.0 |
| Histology differentiation | |  |
| Well |  | 9 |
| Moderate |  | 18 |
| Poor |  | 15 |
| Local invasion | |  |
| Tis-T_2_ |  | 10 |
| T_3_-T_4_ |  | 32 |
| Lymph node metastasis | |  |
| Yes |  | 19 |
| No |  | 23 |
| TNM stage |  |  |
| I-II |  | 9 |
| III-IV |  | 33 |
| Time of follow-up (months) | | 1-34, median=8 |
| TNM, tumour-nodes-metastasis, based on the American Joint Committee on Cancer/International Union Against Cancer Staging Manual (7th edition, 2009) | | |
|  |  |  |
|  |  |  |
