# Supplementary material for: MicroRNA-30a-5p inhibits gallbladder cancer cell proliferation, migration and metastasis by targeting E2F7
Source: Cell Death Dis. 2018 Mar 14;9(3):410. doi: 10.1038/s41419-018-0444-x (PMC5852001; doi:10.1038/s41419-018-0444-x)
Supplement: Supplementary file 6 — supplementary table 2(DOCX 15 kb) [file 41419_2018_444_MOESM6_ESM.docx]

| **Supplementary Table 2. The nucleotides applied in the study** | | |
| --- | --- | --- |
| Description | Name | Sequence |
| Primers for qRT-PCR | hsa-miR-30a-5p | TGACCGATTTCTCCTGGTGTTC |
|  | U6 | ATGGACTATCATATGCTTACCGTA |
|  | E2F7-F | ACCCTCAGATTCCACAGACC |
|  | E2F7-R | AGTTTGCTGTTGCCTTTCCT |
|  | GAPDH-F | AGAAGGCTGGGGCTCATTTG |
|  | GADPH-R | AGGGGCCATCCACAGTCTTC |
| siRNA for E2F7 | siRNA | GACAUCUAGCGCAUCGUCUdTdT |
|  | siE2F7 | ACCCACAGCUAACCUGAAGAUdTdT |
| Sequences for has-miR-30a-5p | Anti-NC | ΜCΜACΜCΜΜΜCΜAGGAGGΜΜGΜGA |
|  | Anti-miR-30a-5p | CΜΜCCAGΜCGAGGAΜGΜΜΜACA |
|  | miR-NC | ΜCACAACCΜCCΜAGAAAGAGΜAGA |
|  |  | ΜCΜACΜCΜΜΜCΜAGGAGGΜΜGΜGA |
|  | miR-30a-5p mimics | ΜGΜAAACAΜCCΜCGACΜGGAAG |
|  |  | CΜΜCCAGΜCGAGGAΜGΜΜΜACA |
